# Aortic distensibility and coronary artery bypass graft patency

**DOI:** 10.1186/1749-8090-4-14

**Published:** 2009-03-26

**Authors:** Bülent Özdemir, Murat Biçer, Levent Özdemir, İbrahim Baran, Aysel Aydın Kaderli, Tunay Şentürk, Ali Emül, Zeynel Abidin Yetgin, Sümeyye Güllülü, Ali Aydınlar

**Affiliations:** 1 Cardiology Department, Uludağ University Medical Faculty, Görükle, Bursa, Turkey; 2 Cardiovascular surgery Department Uludağ University Medical Faculty, Görükle, Bursa, Turkey; 3 Public Health Department, Cumhuriyet University Medical Faculty, Sivas, Turkey

## Abstract

**Background:**

Aortic distensibility is an elasticity index of the aorta, and reflects aortic stiffness. Coronary artery disease has been found to be substantially associated with increased aortic stiffness. In this study we aimed to retrospectively analyze the association of angiographically determined aortic distensibility with the patency rates of coronary bypass grafts

**Methods:**

The study was conducted in the Cardiology department of the Applied Research Centre for Health of Uludağ University. The coronary angiograms of 53 consecutive coronary bypass patients were analysed retrospectively. Aortic distensibility was calculated using the formula: 2 × (change in aortic diameter)/(diastolic aortic diameter) × (change in aortic pressure). The number of stenosed and patent bypass grafts and the patient characteristics like age, risk factors were noted.

**Results:**

There were 44 male (83%) and 9 female (17%) cases. Eighteen cases had only one saphenous vein grafting. The number of cases with two, three and four saphenous grafting were 18, 11 and 1; respectively. In the control angiograms the number of cases with one, two, three and four saphenous vein graft obstruction were 15 (31.3%), 7 (14.6%), 1 (2.1%) and 1 (2.1%) respectively. The aortic distensibility did not differ in cases with and without saphenous graft occlusion (p > 0.05). Also left internal mammary artery (LIMA) graft patency was not related to the distensibility of the aorta (p > 0.05). We also evaluated the data for cut-off values of 50 and 70 mmHg of pulse pressure and did not see any significant difference between the groups in terms of saphenous or LIMA grafts.

**Conclusion:**

In this study we failed to show association of angiographically determined aortic distensibility with coronary bypass graft patency in consecutive 53 patients with coronary artery bypass graft surgery (CABG).

## Background

The long-term patency of the arterial and venous grafts determines the success of coronary artery bypass grafting. Most of the decrease in patency of arterial grafts is associated with moderate stenosis of the native coronary artery and most of the decrease in patency of vein grafts was associated with graft disease itself [1]. Immediate postoperative graft failure may happen and the long-term patency of the saphenous vein graft may be affected by fibro-intimal hyperplasia during the first year after surgery and by atherosclerosis beyond the fifth postoperative year [2,3]. Aortic distensibility is an elasticity index of the aorta, and reflects aortic stiffness [4]. Coronary artery disease has been found to be substantially associated with increased aortic stiffness [5].

In this study we aimed to retrospectively analyze the association of angiographically determined aortic distensibility with the patency rates of coronary bypass grafts.

## Methods

The study was conducted in the Cardiology department of the Applied Research Centre for Health of Uludağ University. Siemens cardiac catheterization unit (an Axiom Artis BC biplane) was used. We aimed to investigate the effects of aortic distensibility on coronary by-pass graft patency. The coronary angiograms of 53 consecutive coronary bypass patients were analysed retrospectively. After Approval from the Research Ethics Committee of Medical Faculty of Uludağ University, we included CABG cases that had coronary angiography between 07 October 2007–07 October 2008. The patient characteristics with the classical coronary risk factors were noted. The angiograms of the cases that were recorded digitally were uploaded to the Siemens Axiom Artis angiography system. The software enabled us to measure the aortic diameters from the uploaded data. All measurements were performed by two investigators. The internal diameters of the aorta in end-diastole and in end-systole were determined at two levels. The first diameter was drawn at the aortic orifice level; and the second was drawn 3 cm above the aortic valve. Systolic and diastolic diameters measured at this level, 3 cm above the aortic valve, were expressed in cm. Aortic distensibility was calculated using the formula: 2 × (change in aortic diameter)/(diastolic aortic diameter) × (change in aortic pressure), where change in aortic diameter = systolic minus diastolic aortic diameter, change in aortic pressure = systolic minus diastolic aortic pressure [6]. Aortic pulse pressure was calculated as systolic aortic pressure – diastolic aortic pressure. Hypertension was defined as having blood pressure greater than or equal to 140/90 mmHg or being on treatment, and diabetes mellitus was defined as having fasting blood glucose >126 mg/dl on two occasions or being on treatment. The reason we used the invasive method for calculation of aortic distensibility was being a gold standard technique and being more easy by enabling us to assess coronary vasculature and distensibility parameters in the same session. Also we think we addressed the use of this nearly forgotten invasive method by cardiologists. Without using echocardiography we can obtain data from the readily derived images during ventriculography and hemodynamic measurements. This also will increase patient compliance for future studies.

The coronary angiograms were evaluated. The number of saphenous and LIMA, RIMA grafts were recorded. The number of patent bypass grafts (with less than %50 angiographical stenosis) was noted. Ki-Square Test was used for classified variables and Mann-Whitney U test for comparison of the means. The statistical analyses were performed by use of SPSS data Manager Software system. Statistical significance was assumed in case of a p value < 0.05. The Results are expressed as mean ± standard deviation.

## Results

### Patient Characteristics and Patency Rates

There were 44 male (%83) and 9 female (%17) cases. The clinical diagnosis was stable angina pectoris in 28 cases, unstable angina pectoris in 22 cases and non ST-elevation in 3 cases. 12 cases (%22.6) were smokers. Native coronary artery disease data and the data of aortic diameter and pressures are given in Table 1. Type 2 Diabetes Mellitus was present in 18 cases (%34). Thirty one cases (%58.5) had hyperlipidemia. Forty (%75.5) cases had hypertension. Eighteen cases had only one saphenous vein grafting. The number of cases with two, three and four saphenous grafting were 18, 11 and 1; respectively. The patient characteristics according to saphenous graft patency results are given in Table 2. In the control angiograms the number of cases with one, two, three and four saphenous vein graft obstruction were 15 (%31.3), 7 (%14.6), 1 (%2.1) and 1 (%2.1) respectively (n = 48). In 24 cases (%50) all the saphenous anastamoses were patent. The number of patients with aorta to left anterior descending artery territory saphenous anastomosis was 24 and 8 (%33.3) of them had occlusion. In 6 cases the anastomoses was to the left anterior descending artery itself and in the remaining patients to the diagonal branches. Of the 34 patients with aorta to circumflex artery territory saphenous anastomosis 11 had (%32.4) occlusions. The number of patients with aorta to right coronary artery territory saphenous anastomosis was 27 and %48.1 had occlusions. Forty eight cases had LIMA anastomosis and 7 (%14.6) were occluded.

**Table 1:** Native coronary lesions and the aortic diameter and pressure values of the whole study patients.

	***Number of Cases***
Number of diseased native coronary vessels (n)	
1 vessel disease	3 (5,7%)
2 vessel disease	15 (28,3%)
3 vessel disease	35 (66%)

	***Mean ± SD***

Systolic aortic Pressure (mmHg)	149,4 ± 27,1
Diastolic aortic Pressure (mmHg)	77,9 ± 13,0
Systolic aortic diameter (cm)	3,29 ±,37
Diastolic aortic Pressure (cm)	3,18 ±,36
Aortic pulse pressure (mmHg)	71,6 ± 21,7
Aortic distensibility (cm2.dyne-1)	4,93 ± 3,3
Years from bypass	8,5 ± 4,8

SD: standard deviation

**Table 2:** Patient Characteristics according to saphenous graft patency

	**Patients with at Least one occluded Saphenous Graft**	**Patients with Patent Saphenous Grafts**	**P value**
Mean Age	63,85 ± 7,69	60,62 ± 9,54	0,215

Gender (M/F)	15/5	18/3	0,454

Smoking	3	6	0,454

Type 2 DM	6	8	0,744

Hyperlipidemia	15	10	0,111

Hypertension	15	16	1,000

Patient Characteristics according to saphenous graft patency. Values are given as mean ± standard deviation in required fields.

### Aortic distensibility and pulse pressure

**Figure 1:**
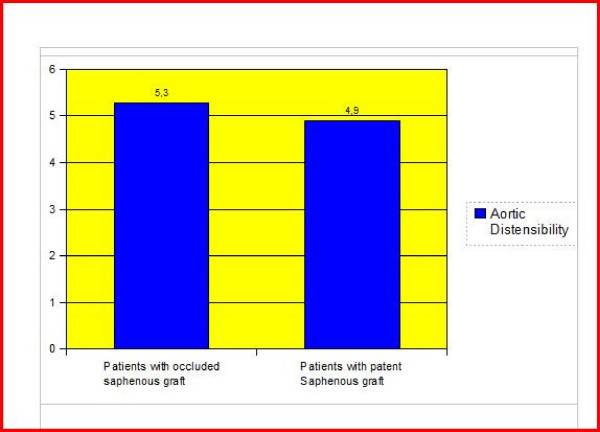
**The comparison of aortic distensibility) in cases with and without saphenous graft**. The comparison of aortic distensibility (cm^2^.dyne^-1^) in cases with and without saphenous graft anastomosis revealed no statistically significant difference (p > 0.05)

**Figure 2:**
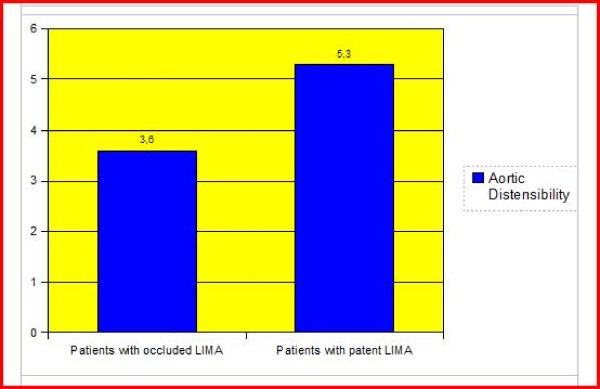
**The comparison of aortic distensibility in cases with and without stenosed left internal mammary artery graft**. The comparison of aortic distensibility (cm^2^.dyne^-1^) in cases with and without stenosed left internal mammary artery graft revealed no significant difference (p > 0.05).

**Figure 3:**
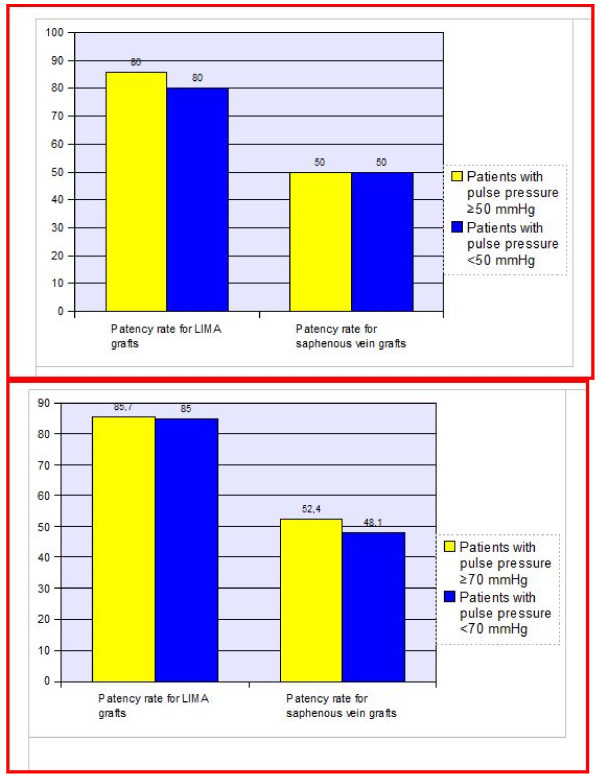
**The percent of saphenous and LIMA grafts stenosis according to aortic pulse pressure**. The percent of saphenous and LIMA graft stenosis was similar in the group with a cut-off value of 50 mmHg (Figure 3-a) and 70 mmHg (Figure 3-b) for aortic pulse pressure (p > 0.05 for both comparisons).

The aortic distensibility did not differ in cases with and without saphenous graft occlusion (p > 0.05) (Figure 1). Also LIMA graft patency was not related to the distensibility of the aorta (p > 0.05) (Figure 2). When the individual anastomoses were analysed (territories of left anterior descending artery, circumflex and right coronary arteries) there was no significant difference in terms of aortic distensibility and pulse pressure. We also had a cut off value of 3.5 (cm2.dyne-1) for aortic distensibility and compared the cases with and without saphenous and/or LIMA graft occlusion. Again there was no statistically significant difference between the groups. A cut-off point for pulse pressure of 50 mmHg was also used for statistical analysis about the patency rates of LIMA and saphenous grafts and there was no statistically significant difference between the groups (p > 0.05) (Figure 3-a). We divided the cases into groups as having aortic pulse pressure lower and higher than 70 mmHg and then checked for patency rates of saphenous and LIMA grafts again. This analysis also did not reveal a statistically significant difference between the groups (p > 0.05) (Figure 3-b).

## Discussion

Cho et al. showed that the patency rates were significantly higher in arterial grafts than in vein grafts at both one year and five years after CABG [1]. Concordantly, in our study, the overall patency rate for LIMA was 85.4% and patency rate for saphenous grafts was 60% (total number of saphenous grafts was 91 and number of occluded saphenous grafts was 36). Goldman et al reported similar results. The 10-year patency in his study was 61% for SVGs and 85% for IMA grafts in a study by Goldman [7].

In our study in cases with and without saphenous graft obstruction; the mean number of years passed since bypass were 8.9 ± 5.6 and 7.8 ± 4.3; respectively, being significantly indifferent. Cases with patent and occluded LIMA had a mean number of years passed since bypass of 9.2 ± 4.8 and 5.7 ± 1.9; respectively (p > 0.05). Campeau et al. observed that elevated plasma cholesterol and LDL as well as low HDL were associated with atherosclerotic progression in grafts [8]. Daida et al. found a strong relationship of cholesterol level and vein graft obstruction in 284 patients a mean of seven years after bypass [9]. In a study it was reported that post-CABG patient and graft characteristics associated with SVG atherosclerosis progression including: maximum stenosis of the graft at baseline angiography; years post-SVG placement; the moderate LDL-C lowering strategy; prior MI; high triglyceride level; minimum graft diameter; low HDL cholesterol; high LDL cholesterol; high mean arterial pressure; low ejection fraction; male gender; and current smoking [10].

Many studies have shown that arterial stiffness is the most important cause of cardiovascular complications and a major contributor to atherosclerosis, and thus to stroke and myocardial infarction [11]. Decreased distensibility (a stiffer aorta) was associated with older age, hypertension, and African American ethnicity. Additionally, decreased aortic distensibility was present in current smokers and in subjects with higher HDL cholesterol levels [12].

In prior studies it has been shown that pulsatile changes in ascending aortic diameter can be measured during routine transthoracic echocardiography. Stefanadis et al. described that noninvasive measurements of aortic distensibility by echocardiography, based on aortic dimensions and blood pressure data, are as accurate as invasive methods. Impaired elasticity indexes of aorta were correlated with the presence and severity of coronary artery disease as said by Yıldız et al [13].

Aortic atherosclerotic plaque and systolic blood pressure is associated [14]. Associated coronary artery disease and impaired elasticity indexes of aorta might be the manifestations of common atherosclerotic process in addition to the arterial wall nutrition hypothesis [15,16].

In a study by Cay et al., having higher aortic pulse pressure increased the risk of saphenous vein graft occlusion by 3-folds (95% CI: 1.40–6.43) during the early period after CABG [17]. In contrast to Cay et al. [17], the saphenous graft patency rates were similar in cases with aortic pulse pressure lower than 50 mmHg compared to cases with pressures 50 mmHg or above in our study. In his study there was 126 patients and also the cut-off point was 50 mmHg for aortic pulse pressure. Our results are reflective of late term results for coronary saphenous grafts. Analysis of the data with a cut-off value of 70 mmHg for pulse pressure also revealed no significant difference in terms of LIMA or saphenous graft patency (Figure 3-b). However only pulse pressure itself may be far from giving clue about the aortic properties. As an elasticity index, distensibility which uses aortic pulse pressure as a denominator of the formula provides more concise result. The pulse pressure in our study was high though the age of the study group was comparable to the group in the study of Cay et al [17]. However the blood pressures in our group was higher than the normal values. This may have caused the observed increase in aortic pulse pressure. Furthermore; the elasticity of the aorta did not show the expected variation among patients with and without saphenous vein and the LIMA grafts. Also in our clinic in some cases benzodiazepines are used for sedation and the effects of these drugs in aortic elastic properties and blood pressure may have affected the results.

## Conclusion

In this study we failed to show association of angiographically determined aortic distensibility with coronary bypass graft patency in consecutive 53 bypass patients. However; this was a retrospective study with considerably small study sample. Prospective Studies with larger numbers, studies taking patency rates at defined intervals into account may provide better understanding of the issue.

## Competing interests

The authors declare that they have no competing interests.

## Authors' contributions

BÖ and MB had helped with design of the study, data interpretation and in writing of the paper. LÖ has made the statistical analysis and took part in the writing process. He also took part in the correction of the manuscript according to the reviewers suggestions. İB, SG and AA helped in writing of the paper. AE and ZAY had helped in gathering patient information and performed angiographic measurements. TŞ and AAK performed graphics and tables and added comments to the paper. All authors read and approved the final manuscript.
